# Effect of Motor Imagery in Children with Unilateral Cerebral Palsy: fMRI Study

**DOI:** 10.1371/journal.pone.0093378

**Published:** 2014-04-09

**Authors:** Eva Chinier, Sylvie N’Guyen, Grégoire Lignon, Aram Ter Minassian, Isabelle Richard, Mickaël Dinomais

**Affiliations:** 1 LUNAM; Université d’Angers, Laboratoire d’Ingénierie des Systèmes Automatisés (LISA), Nantes, France; 2 LUNAM, Université d’Angers, Département de Médecine Physique et de Réadaptation, Nantes, France; 3 LUNAM Université d’Angers, CHU Angers, département de neuropédiatrie, Nantes, France; 4 LUNAM Université d’Angers, CHU Angers, Pôle d’imagerie, Nantes, France; 5 LUNAM Université d’Angers, CHU Angers, Pôle d’anesthésie réanimation, Nantes, France; University of Surrey, United Kingdom

## Abstract

**Background:**

Motor imagery is considered as a promising therapeutic tool for rehabilitation of motor planning problems in patients with cerebral palsy. However motor planning problems may lead to poor motor imagery ability.

**Aim:**

The aim of this functional magnetic resonance imaging study was to examine and compare brain activation following motor imagery tasks in patients with hemiplegic cerebral palsy with left or right early brain lesions. We tested also the influence of the side of imagined hand movement.

**Method:**

Twenty patients with clinical hemiplegic cerebral palsy (sixteen males, mean age 12 years and 10 months, aged 6 years 10 months to 20 years 10 months) participated in this study. Using block design, brain activations following motor imagery of a simple opening-closing hand movement performed by either the paretic or nonparetic hand was examined.

**Results:**

During motor imagery tasks, patients with early right brain damages activated bilateral fronto-parietal network that comprise most of the nodes of the network well described in healthy subjects. Inversely, in patients with left early brain lesion brain activation following motor imagery tasks was reduced, compared to patients with right brain lesions. We found also a weak influence of the side of imagined hand movement.

**Conclusion:**

Decreased activations following motor imagery in patients with right unilateral cerebral palsy highlight the dominance of the left hemisphere during motor imagery tasks. This study gives neuronal substrate to propose motor imagery tasks in unilateral cerebral palsy rehabilitation at least for patients with right brain lesions.

## Introduction

Cerebral Palsy (CP) is a major cause of motor disability in children. Among these CP children, approximately one third have hemiplegic cerebral palsy (HCP, unilateral CP) which is a form of CP caused by unilateral lesion on a developing brain and is characterized by a unilateral motor impairment. Limitations in movement of paretic hand significantly reduce its effective use in daily life activities [1]. Recent studies highlighted that daily life difficulties in HCP patients are not only due to motor execution problems on their affected side but also to motor planning disabilities on both sides [2]. Motor planning is defined as a cognitive process in which the patient prepares and executes a series of appropriate movement toward an object [3] while taking into account the immediately available information and the forward internal model [4]. The ability to form internal representations of a motor act is dependent of the motor experience and motor skills development [5], [6].

Although, there is no cure for hemiplegia, its effects can be minimized through neurorehabilitative approaches. Current rehabilitation interventions to treat motor impairments are mainly based on techniques aiming at stimulating repetitively the use of the paretic limb during supervised sessions (for a review, see [7]). However, few rehabilitation approaches are intended to enhance motor preparation or motor planning abilities. Thus, new tools of CP rehabilitation that focus on motor planning disabilities have been proposed.

One potentially useful rehabilitation intervention targeting cognitive aspects of motor behaviors may be motor imagery (MI) [8]. MI consists for a subject in imagining the execution of a simple or complex movement that is not accompanied by bodily motion, without any other movement. Thus, MI is an active cognitive process in which the action representation is internally reproduced within the working memory, without overt execution [9], [10] in a way that is similar to when the subject actually performs movement [11]. A growing number of neuroimaging studies have demonstrated that many of the brain structures (for example, pre-supplementary motor area (pre-SMA), dorsal premotor cortex (dPMC), supramarginal gyrus, superior parietal lobe (SPL), cingulate cortex, and cerebellar regions) involved in performing real movements also show increased activity during imagined movements [12] with a predominance in left hemisphere [13].

Using MI as a therapy to enhance the effects of motor rehabilitation in patients with unilateral CP raises at least two questions: 1) Do MI tasks engage brain activation despite the existence of early brain lesion? and 2) Do MI tasks engage similar brain activation in cases of left and right early lesions located in the brain motor system? Indeed, in 2011, Williams and al. [14] hypothesized that motor planning deficit in HCP patients may stem from poor motor imagery ability as they are both based on internal representation of movement. More, because of its concealed nature, the ability to perform motor imagery in HCP is difficult to assess. Until now, only neuropsychological studies tried to evaluate the ability for HCP patients to perform motor imagery but gave no conclusive data [5]. To our knowledge, no functional imagery study has been done on MI in HCP patients.

The aim of this study was to examine brain activations in HCP patients following MI tasks, and compare brain activation induced by MI depending on whether the patient imagined a hand movement performed by his nonparetic hand (nph), or by his paretic hand (ph). Finally we assessed the effect of the side of the lesion on brain activation.

## Materials and Methods

### Method

#### Participants

Twenty children (sixteen males, mean age 12 years and 10 months, aged 6 years 10 months to 20 years 10 months) with clinical apparent congenital hemiparesis and no clinical arguments [15] for contralesional hemisphere reorganization of motor cortex were included (Table 1). Subjects 1 to 18 were recruited as part of our previous work investigating the action-observation network in patients with CP [16], [17]. Subjects 19 and 20 with clinical apparent left unilateral CP were added in this study. Ten subjects had radiological evidence of an involvement of unilateral central regions in the lesion and 10 subjects had no cortical involvement but unilateral periventricular white matter lesion. The population analysed here are constituted from 11 subjects suffered from right hemiparesis (left brain lesions) and 9 from left hemiparesis (right brain lesion). Approval was given by the local Ethics Committee (Comité de protection des personnes, CPP ouest II, Angers, France, n° A.C = 2011-AO1056-35, n°CPP: 2011/29). All subjects and parents gave written, informed consent. Exclusion criteria were as follows: severe mental retardation, severe vision impairment, severe attention disorders and presence of mirror movements [15].

**Table 1:** Demographic data.

Subjects	Sex	Age at fMRI (year)	Lesion side (L/R)	Hemiparesis side (L/R)	Lesion type	Hand motor dysfunction (1–4)	BFMF (1–5)
1	M	16	L	R	PVL	2	1
2	F	12	L	R	MCA	2	1
3	M	10	L	R	MCA	3	2
4	M	9	L	R	MCA	2	1
5	M	13	L	R	MCA	4	3
6	M	11	L	R	PVL	2	1
7	M	12	L	R	MCA	2	1
8	M	20	L	R	MCA	3	2
9	M	21	L	R	MCA	4	3
10	M	10	L	R	PVL	2	1
11	M	16	L	R	PVL	3	2
12	M	15	R	L	PVL	3	2
13	M	11	R	L	PVL	2	1
14	F	18	R	L	PVL	3	2
15	F	15	R	L	PVL	2	1
16	M	16	R	L	MCA	3	2
17	M	6	R	L	MCA	2	1
18	F	7	R	L	MCA	2	1
19	M	10	R>L	L	PVL	2	1
20	M	9	R>L	L	PVL	2	1

*Note*: L = Left; R = Right; PVL = Periventricular Lesion; MCA = Middle Cerebral Artery Stroke; BFMF = Bimanual Fine Motor Function.

The Bimanual Fine Motor Function (BFMF) [18] was used to categorize children with unilateral CP into five levels according to their ability to perform fine motor function in bimanual activities. The contralesional hand function (paretic hand) was also assessed with the sequential finger opposition task as described previously by Staudt et al. [19]: 1 =  normal performance, 2 =  slow performance or incomplete performance, 3 =  inability to perform any independent finger movement, but with a preserved grasp function, and 4 =  no active grasping.

### Materials and Procedures

#### Experimental paradigm

For functional MRI acquisition, the paradigm was implemented in block designs, with two conditions: 1) Patients were asked to imagine that they performed an opening-closing hand movement at a frequency of 1,5 Hz guided by the sound of a “metronome” (1.5Hz) (auditory-paced MI tasks) [20]. During MI task, subjects saw a screen displaying a fixed hand of an actor from the third person perspective on the same side than the MI task. This vision MI task is reported thought [21] to improve performance by facilitating motor representation. During MI tasks, patients were instructed not to make any voluntary movement. The patient received no further instruction regarding the modality for MI (1^st^ or 3^rd^ person). The absence of actual hand movement during the motor imagery task was visually controlled by the investigator. This condition represented the motor-imagery condition (MI condition). 2) Patients at rest watched a black screen with a fixed red cross in the centre. This latter condition represents the baseline condition (RES condition). During one session, each task was performed for twenty one seconds and repeated six times.

For each participant, two independent functional sessions were performed, one session, where the participant imagined moving his paretic hand (“paretic hand (ph) MI”), and one session where the participant imagined moving his nonparetic hand (“nonparetic hand (nph) MI”). For example, in the “paretic hand MI session (MI_ph_)”, a patient with left unilateral CP imagined a left opening-closing hand movement at 1.5 hz and in the “nonparetic hand MI session (MI_nph_)”, he imagined a right opening-closing hand movement at 1.5 hz.

Before fMRI scanning, all subjects received detailed instructions and were trained for the two motor-imagery tasks with a metronome. When all subjects reported that they could easily perform the motor imagery task for both hands, the MRI acquisition could begin. At the end of MRI sessions, all participants declared that they did well the MI tasks in both sessions.

#### Data acquisition

All datasets were acquired on a 1.5 T MR scanner (Magnetom Avanto, Siemens Medical Systems, Erlangen, Germany). An EPI sequence was used to acquire functional series in each subject (TR = 3000 ms, TE = 50 ms, flip angle = 90°, 32 axial interleaved slice of 5 mm slice thickness, in plane matrix = 64×64 with a field of view = 240 mm, yielding a voxel size of 3×3×5 mm^3^), covering the whole brain including the cerebellum. We acquired 84 functional volumes per session over two sessions. A T1-weighted anatomical 3D data set was also obtained, covering the whole brain (176 contiguous sagittal slices, in-plane matrix 256×256, yielding a voxel size of 1×1×1 mm^3^).

#### Analysis of imaging data

The image data were analysed using SPM8 (Wellcome Department of Imaging Neuroscience, University College, London, UK, http://www.fil.ion.ucl.ac.uk/spm) and in part, by custom routines. Firstly, as brain lesions were either on the left or on the right side of the brain, images of subjects with right lesion were flipped so that all lesions were on the left side of the images. Then, all images were realigned to the first image of the first session and unwarped to correct hand movement [22]. The 3D-dataset was segmented in native-space, using a unified segmentation approach [23] and then coregistered to the mean functional image from the first session. Segmentation parameters were used to spatially normalize data into a standard stereotaxic space with a final resolution of 3×3×3 mm. The crucial step of normalization capitalizes on the fact that during tissue segmentation, chronic lesions are overwhelmingly classified as CSF [24]. This tissue class is then used as the basis for an automatically-generated lesion mask which in turn is used to implement a cost-function masking approach [25] during spatial normalization. This normalization procedure was successfully already applied to subjects with such brain lesions [16], [17]. Finally, the images were spatially smoothed 8 mm full width at half-maximum [FWHM].

Using the REST_black_ condition as tailored baselines, for each session (MI_ph_ or MI_nph_) individual contrast images for the contrasts MI > REST were used in second-level random effects analysis. A whole brain random-effects full 2 (MI conditions) ×2 (side of brain lesion) ANOVA was conducted on the fMRI data, with the side of imagined hand movement (imagination side, ph or nph) as within-subject factor and side of the lesion (right or left) as between-subject factor. This statistical analysis allows to test for potential differences between MI of the nph and MI of the ph and the influence of the side of the lesion. Thus, Statistical F-maps were created for each main effect and for each interaction, thus the general motor imagery network across all conditions was determined using an F-contrast. Because F-maps do not contain information about the direction of the main effects, statistical t-contrasts were calculated to determine the direction of any significant main effects.

All statistical parametric maps were interpreted after applying a threshold of p<0.05 corrected for multiple comparisons. The correction for multiple comparisons was performed using Monte Carlo simulation determined by AlphaSim program in the Resting-State fMRI Data Analysis Toolkit V1.7 (http://www.restfmri.net/forum/index.php). Anatomical labels were ascribed to the activation maxima using the Anatomy toolbox [26].

## Results

### Main Effect of Motor Imagery

**Figure 1:**
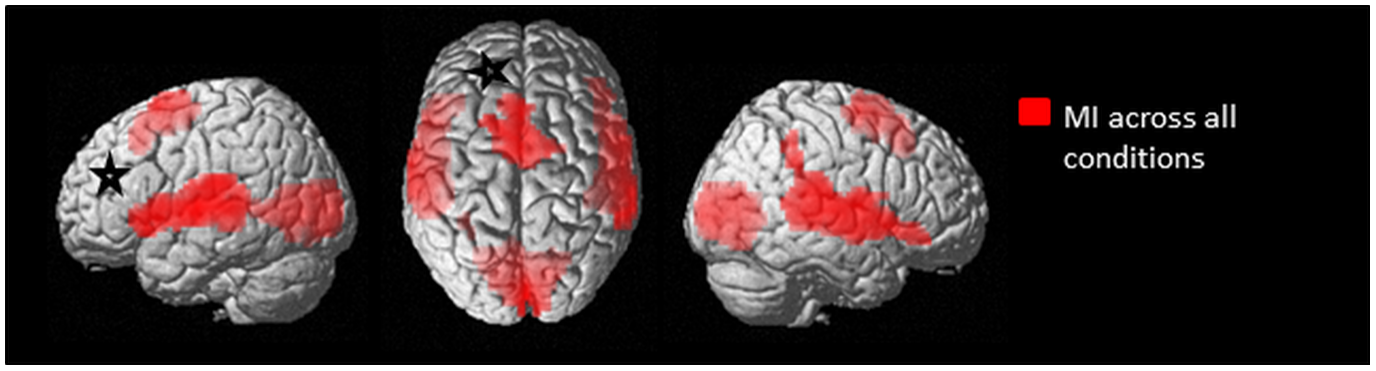
Statistical maps illustrating the brain activations during motor imagery tasks across all conditions. The results are superimposed in red on a standard rendered single subject brain (available on SPM8). Results are showed with a significance of p<0.05 corrected for multiple comparisons (alpha-simulation). The black and white stars indicate schematically the ipsilesional hemisphere. *Notes*: MI, motor imagery.

Coordinates for all peaks of brain activations following MI when averaged across the side of lesions (left or right) and the side of imagined hand movement (ph or nph) can be found in Table 2 (see also Fig. 1). MI activates large bilateral clusters. In the frontal lobes, significant activations were found in bilateral inferior frontal gyrus (pars orbitalis, pars opercularis and pars triangularis), in bilateral pre-supplementary motor area (pre-SMA) (Brodman Area, BA6) and in bilateral superior frontal gyrus. In the temporal lobe, bilateral superior temporal gyrus, contralesional temporal pole and ipsilesional middle temporal gyrus were activated. In the parietal lobes, significant activations were found in bilateral supramarginal gyrus (SMG) (parietal operculum, especially OP1) and contralesional inferior parietal gyrus. In the occipital lobe, ipsilesional calcarine gyrus and bilateral lingual gyrus (BA17) were activated.

**Table 2:** Peaks of consistent activation across all conditions.

				MNI coordinates		
Region	Side	Cluster size	*t*-Value	x	y	z
Calarine gyrus	IL	1413	6.78	3	−87	3
Lingual Gyrus	IL		4.40	−21	−66	0
	CL		4.25	18	−72	0
	IL		4.03	−12	−60	3
Superior Temporal Gyrus	CL	904	7.71	60	−15	6
	CL		7.71	54	0	0
	CL		4.91	51	−6	−6
	CL		4.58	66	−27	18
Temporal Pole	CL		4.52	54	6	−3
Superior Temporal Gyrus	CL		4.10	45	−33	0
	CL		3.52	48	−24	−3
Inferior Frontal Gyrus	CL		3.39	51	27	−6
	CL		3.35	45	36	−12
SupraMarginal gyrus	CL		3.30	63	−42	36
Inferior Parietal Gyrus	CL		2.91	57	−42	51
Superior Temporal Gyrus	IL	899	7.29	−63	−12	3
SupraMarginal gyrus	IL		5.29	−60	−24	21
Inferior Frontal Gyrus	IL		5.00	−51	21	−6
Superior Temporal Gyrus	IL		4.94	−63	−27	6
Middle Temporal Gyrus	IL		4.84	−57	−36	6
Inferior Frontal Gyrus	IL		4.60	−51	9	6
	IL		4.56	−57	12	3
Superior Temporal Gyrus	IL		4.07	−45	−18	3
Middle Temporal Gyrus	IL		4.02	−48	−30	3
Superior Temporal Gyrus	IL		3.87	−48	−21	6
Inferior Frontal Gyrus	IL		2.88	−39	24	3
Supplementary motor area	IL	404	5.10	0	6	60
	IL		3.90	−6	21	45
	IL		3.65	0	−6	63
	IL		3.26	−6	3	72
	IL		3.09	−12	9	66
	CL		3.06	15	−3	66
Superior Frontal Gyrus	IL		2.89	−21	15	66
	IL		2.85	−18	12	63
Supplementary motor area	CL		2.69	6	−6	54
Superior Frontal Gyrus	CL		2.52	24	−3	57

*Notes*: CL, contralesional hemisphere; IL, ipsilesional hemisphere.

p<0.05 corrected for multiple comparisons (alpha-simulation).

### Main Effect of Imagination Side

**Figure 2:**
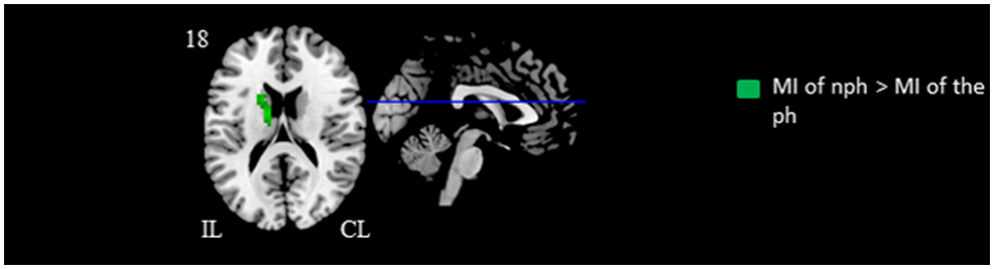
Statistical maps illustrating the influence of the side of imagined hand movement. Motor imagery of nonparetic hand movement > motor imagery of paretic hand movement is represented in red color. The black and white stars indicate schematically the ipsilesional hemisphere. Results are showed with a significance of p<0.05 corrected for multiple comparisons (alpha-simulation). *Notes*: CL, contralesional hemisphere; IL, ipsilesional hemisphere; nph, nonparetic hand; ph, paretic hand; MI, motor imagery.

Only the ipsilesional caudate nucleus showed significant higher activation when the subject imagined movement of his nph compared to when subject imagined movement of his ph (Figure 2, Table 3). Inversely, there were no significant suprathreshold voxels when the subject imagined movement of his ph compared to when subject imagined movement of his nph.

**Table 3:** Peaks of consistent during motor imagery of the unaffected hand compared with motor imagery of the affected hand.

				MNI coordinates		
Region	Side	Cluster size	*t*-Value	x	y	z
Caudate Nucleus	IL	92	4.49	−12	−9	21
	IL		3.74	−21	12	21
	IL		3.42	−18	9	18
	IL		2.73	−15	15	12

*Notes*: CL, contralesional hemisphere; IL, ipsilesional hemisphere.

p<0.05 corrected for multiple comparisons (alpha-simulation).

### Main Effect of the Side of Brain Lesion

**Figure 3:**
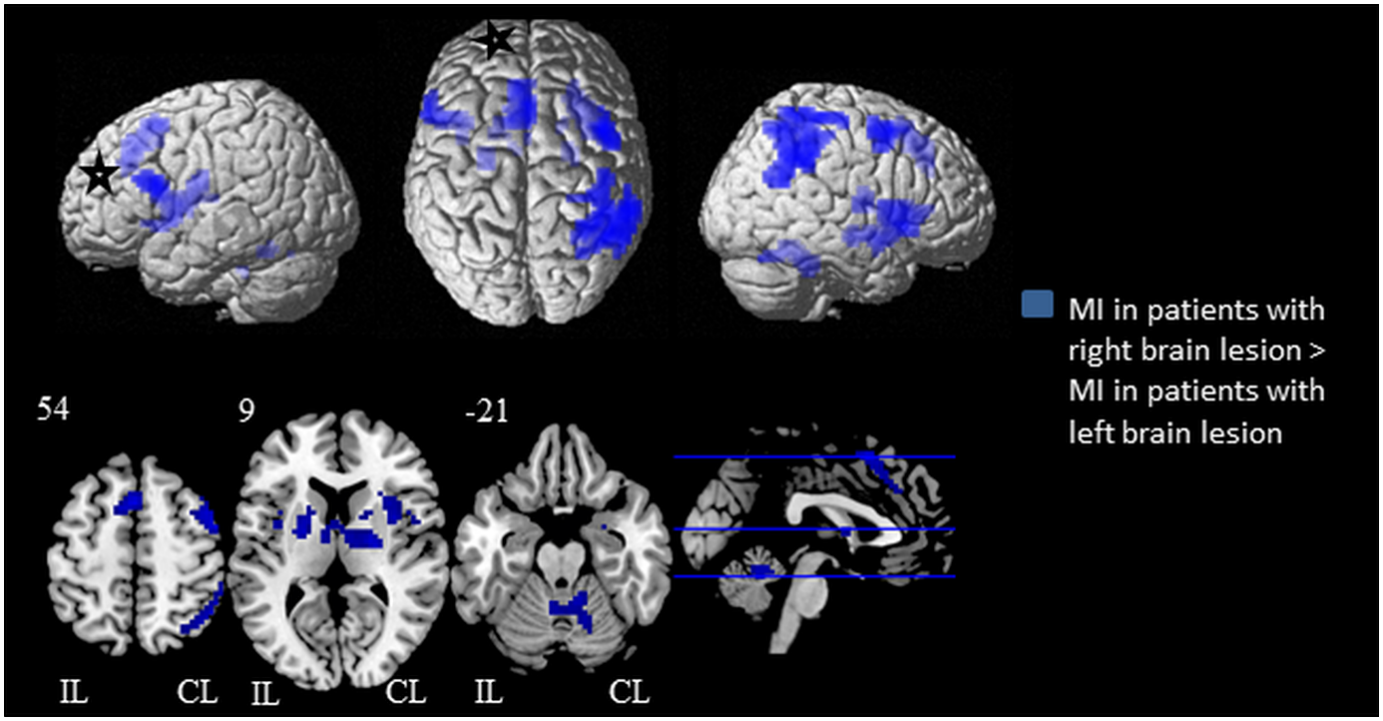
Statistical maps illustrating the influence of brain lesion’s side on brain activations following motor imagery tasks. Brain activations in subjects with right early brain lesion > subjects with left early brain lesion is superimposed in red on a standard rendered single subject brain (available on SPM8). Results are showed with a significance of p<0.05 corrected for multiple comparisons (alpha-simulation). The black and white stars indicate schematically the ipsilesional hemisphere. *Notes*: CL, contralesional hemisphere; IL, ipsilesional hemisphere; nph, nonparetic hand; ph, paretic hand; MI, motor imagery.

When subjects with right brain lesion performed MI tasks compared to when subjects with left lesion performed the same MI tasks, several brain regions showed significant higher activations (Figure 3, Table 4). Thus, we found significant activations in ipsilesional pre-SMA, in ipsilesional superior frontal medial gyrus, in ipsilesional inferior frontal gyrus (pars opercularis), in contralesional precentral gyrus, in contralesional middle frontal gyrus (BA6) and in in the ipsilesional insula lobe. In the temporal lobe, contralesionall Rolandic operculum (especially OP4) was also higher activated. In the parietal lobes, significant higher activations were found in contralesional angular gyrus, in contralesional superior parietal lobule, in contralesional inferior parietal gyrus, in contralesional supramarginal gyrus and in contralesional postcentral gyrus (BA6). In the subcortical region, contralesional hippocampus, bilateral putamen, bilateral thalami were higher activated. In cerebellar area, bilateral cerebellum (lobule I-IV) and cerebellar vermis were consistently more activated.

**Table 4:** Peaks of consistent during motor imagery by patients with right lesion compared to motor imagery by patients with left brain lesion.

				MNI coordinates		
Region	Side	Cluster Size	*t*-Value	x	y	z
Hippocampus	CL	413	4.10	18	−15	−12
Putamen	CL		3.95	30	15	6
	CL		3.15	18	12	3
	CL		2.94	21	9	6
Rolantic Operculum	CL		2.59	48	6	9
Angular Gyrus	CL	392	4.20	57	−57	30
Superior Parietal Lobule	CL		4.03	33	−54	66
Inferior Parietal Lobule	CL		3.92	57	−51	39
Postecentral Gyrus	CL		3.78	45	−42	63
SupraMarginal Gyrus	CL		3.53	57	−42	36
Inferior Parietal Lobule	CL		3.36	48	−54	54
	CL		3.23	42	−57	57
	CL		3.22	57	−42	51
	CL		3.20	57	−36	51
Postcentral Gyrus	CL		3.19	36	−30	60
Angular Gyrus	CL		3.11	42	−51	36
Thalamus	CL	267	3.70	21	−9	6
Putamen	IL		3.57	−21	3	6
	IL		3.48	−21	−3	9
Thalamus	CL		3.26	6	−9	6
	IL		3.22	−9	−3	12
Putamen	IL		3.03	−18	12	−6
	IL		2.75	−30	3	−3
	IL		2.65	−12	−15	12
Suplementary Motor Area	IL	141	3.98	0	15	54
	IL		3.76	−6	12	57
Superior Medial Gyrus	IL		3.62	−6	24	42
	IL		3.55	−6	30	36
Cerebellum	IL	137	3.53	−3	−48	−18
Cerebellar Vermis	CL		3.36	6	−51	−18
Cerebellum	CL		2.96	15	−57	−21
	CL		2.63	24	−69	−24
Inferior Frontal Gyrus	IL	99	3.89	−51	12	18
	IL		3.48	−39	12	15
Insula Lobe	IL		2.87	−39	0	9
Precentral Gyrus	CL	74	3.84	42	6	51
Middle Frontal Gyrus	CL		3.72	48	0	54

*Notes*: CL, contralesional hemisphere; IL, ipsilesional hemisphere.

p<0.05 corrected for multiple comparisons (alpha-simulation).

Inversely, there were no significant suprathreshold voxels when subjects with left brain lesion performed MI tasks compared to when subjects with right lesion performed the same MI tasks.

Taken together these results indicate that MI tasks in patients with left early brain lesion activated relatively fewer brain regions.

### Side of Brain Lesion x MI Conditions Interaction

There were no significant activation clusters in the MI conditions (ph or nph) x side of brain lesion interaction, indicating that the effect of the side of brain lesion on brain activation was the same in both side of imagination (MI ph or MI nph).

## Discussion

Our study revealed that MI tasks in patients with unilateral CP engage brain activations despite the existence of motor impairment. Whereas the side of imagined hand movement (nph or ph) had weak influence on brain activations following MI, the side of brain lesion appears crucial. During MI tasks, patients with early right brain lesion activated bilateral fronto-parietal network that comprise most of the nodes of the network well described in healthy subjects [27]. Inversely, in patients with left early brain lesion, we found fewer brain activation following MI tasks compared to patients with right brain lesions. This indicates that lesion on left hemisphere in patient with unilateral CP affect brain activations following MI tasks as described in adult stroke patient with lesion in left hemisphere. Indeed, several studies reported in left brain injured stroke patients with parietal lesion, impairment of MI abilities [28]–[31]. These results shall now be discussed in more detail.

### Differences in Brain Activations between Motor Imagery with the Paretic Hand and with the Nonparetic Hand

Brain activations following MI tasks seem to be similar whatever the side of the imagined hand. Compared to MI with the ph, only the ispilesional nucleus caudate is significantly more activated during the MI with the nph. Inversely, there was no significant activation when we compared MI with the ph to MI with the nph. These results indicate that the side of imagined hand has a weak influence on brain activations following MI. One question raises from these results: Did our patients properly imagine a MI of the ph during the MI_ph_ condition? Or did they instead imagine a MI of the nph? It is well established that patients with unilateral CP have bilateral motor planning disabilities [32]–[35] and present disabilities in internal representation of a movement with both hands (nph or ph). Subsequently during MI, it must be difficult for a patient with unilateral CP to distinguish with vividness the side of the moved hand. In our point of view, during MI, our patients performed MI tasks with a blurry distinction of the side of imagined hand. This could explain the weak influence of imagination side on brain activation.

### Crucial Influence of the Lesion Side on Brain Activation following MI Tasks

While the side of imagined movement seems to have little influence on brain activation following MI tasks, the lesion side influences dramatically the brain activation with poor activation in case of left early brain lesion. This result is well in line with findings in different fields. Neuropsychological studies established that planning motor deficit are more severe in right hemiplegia (left brain damages) [3], [14], [33], [36], [37]. This finding corroborates neuroimaging studies that showed the dominance of the left hemisphere in motor planning [38]–[41]. Mutsaarts and al. [42] established that HCP patients with left brain lesions appear to be unable to mentally simulate movements of either hands whereas patients with right brain damages appear to have more difficulties to mentally simulate movements with their ph than with their nph. However, there are contradictory studies and no conclusive data about the influence of lesion side on mental ability are available [43], [44]. Thus, our study indicates that patients with left early brain injury could be having difficulties to perform MI tasks.

In patients with right early brain lesion, we found a brain network following MI in both MI conditions (ph or nph) which seems to be pretty similar to those described following MI in healthy people. Hétu and al [27] in an ALE meta-analysis of the neural substrate of MI in healthy patients, showed that the meanly brain areas significantly activated were the bilateral fronto-parietal network involving the premotor cortex, the pre-SMA and inferior and superior parietal lobules but also subcortical and cerebellar regions. A large parietal superior activation during MI is well described in numerous fMRI studies and is in accordance with neuropsychological and Transcranial magnetic stimulation (TMS) studies which demonstrated the role of superior parietal region in motor visuo-spatial transformations [45], [46], [47], [48]. It should be noted that in our sample we did not find any activation in primary motor cortex (M1) following MI tasks. The activation of M1 following MI task are matter of debate and to date there is no conclusive data on the involvement of the primary motor cortex following MI (for more details see, [27]).

### Is Motor Imagery, a Promising Rehabilitation Tool in HCP Patients?

There is a growing issue to provide a better understanding of MI and MI abilities in children with CP. Indeed, MI can be used as a ‘backdoor’ access to evaluate the motor system [49] or the neural representation of movement without potential confounds related to sensory feedback and motor output. MI can also be used as a rehabilitation tool called ‘mental imagery practice process’, which consists of repeated imagined motor acts with the intention of improving physical execution [50]. Mental imagery practice process can be used to learn complex motor skills like sports [51] or to re-learn motor skills and enhance motor recovery of motor execution in populations with neurological impairments. It has recently been showed that motor imagery enhances cortico-spinal tract excitation in stroke patients [52]. The interest of mental imagery practice as a rehabilitation tool is established for example in strokes patients [53], and in children with developmental coordination disorders [54].

The use of MI in CP rehabilitation remains a debated subject. Crucial issues stay unanswered like: Does mental imagery practice enhance motor planning in patients? Could MI training be applied to all patients with CP? Our study gives a first response. Regarding to the higher brain activations following MI in patients with right early brain lesions, compared to those with left early brain lesions, MI practice may be interesting in motor planning disabilities in patients with left unilateral CP (right brain lesion).

### Possible Limitations of Our Study

Our population was relatively homogeneous in regards to patients’ clinical assessment (HCP patients without mirror movement). However they differed by the localization of the lesion (periventricular lesion and middle cerebral artery infarct). The design of the study and the use of flipped functional images did not allow a more precise analysis of our data, in order to determine why some areas are activated only on one side. In addition, at least two different modalities of imagery can be described [55]: (1) the subject produces a visual representation of the movement, this is also known as visual imagery (VI); (2) the subject carries out a mental simulation of the movement, which is associated with kinesthetic sensations (this requires to imagine “feeling” the movement), this is also known as kinesthetic imagery. The mixed design of MI tasks (auditory-paced and “vision” MI tasks) used in our study did not allow to discriminate brain activation between these two modalities of MI. However the focus of this study was the description of brain areas involved in MI tasks of in a simple motor task with the hand in CP patients which has, to our knowledge, not been studied to date.

## Conclusion

This fMRI study might shed new light on motor imagery abilities in HCP patients. Showing few activations following MI in patient with right unilateral CP, we highlighted the dominance of the left hemisphere during MI tasks as described in literature. More, we showed few differences between activations during MI tasks of the nph and ph. Thereby, to our opinion, MI practice may be interesting in motor planning disabilities at least in HCP patients with right brain lesion.
